# Biologger-based monitoring of body temperature, heart rate, and heart rate variability in Lidia cattle: relationships with environmental conditions

**DOI:** 10.1007/s00484-026-03199-0

**Published:** 2026-04-15

**Authors:** José-Alfonso Abecia, Pablo Iglesias, Javier Plaza, Jaime Nieto, Luis Morales, Carlos Palacios, Marta Elena Alonso, Juan Manuel Lomillos

**Affiliations:** 1 Facultad de Veterinaria, Instituto Universitario de Investigación en Ciencias Ambientales (IUCA), Universidad de Zaragoza, C/ Miguel Servet. 177, Zaragoza, 50013 Spain; 2 Departamento de Producción Animal, Facultad de Veterinaria, Universidad de León, Campus de Vegazana, S/N, León, 24071 Spain; 3 Área de Producción Animal, Facultad de Ciencias Agrarias y Ambientales, Universidad de Salamanca, Avenida Filiberto Villalobos, 119, Salamanca, 37007 Spain; 4 Departamento de Producción y Sanidad Animal, Salud Pública Veterinaria y Ciencia y Tecnología de los Alimentos, Facultad de Veterinaria, Universidad CEU Cardenal Herrera, C/ Tirant lo Blanc, 7, Valencia, 46115 Spain

**Keywords:** Lidia cattle, Biologgers, Body temperature, Heart rate, Heart rate variability, Circadian rhythm, Heat stress, Thermoregulation, Resilience

## Abstract

An understanding of how Lidia cattle (*Bos taurus)* physiologically adapt to natural environmental conditions is essential for assessing their welfare and resilience in extensive systems. This study documented the circadian and short-term responses of Lidia cows to natural summer conditions based on data from subcutaneous biologgers that continuously recorded body temperature (T), heart rate (HR), and HR variability (HRV). Four cows were monitored for 10 consecutive days, and ambient T and relative humidity were recorded simultaneously, which were used to calculate the Temperature–Humidity Index (THI). Both T and HR exhibited clear diurnal rhythms, increasing during the day and peaking in the evening, but HRV indices (SDNN and RMSSD) were highest at night, which reflected highest parasympathetic activity at night. Cosinor analyses confirmed strong rhythmicity in body T (robustness = 0.84; circadian index > 3) and moderate rhythmicity in HR and HRV. Physiological responses to heat stress exhibited distinct temporal delays; i.e., HR increased approximately 3 h after increases in THI, body T peaked after about 4.5 h, and HRV reached its minimum 3.5–4 h later. Those results indicate that autonomic and thermal adjustments unfolded gradually and with measurable lags following changes in environmental conditions. Despite pronounced daily variations in ambient conditions, Lidia cattle maintained stable body T rhythms and clear circadian organization, which reflected their high thermotolerance and capacity to adjust to Mediterranean summer environments.

## Introduction

The study of animal physiology under natural environmental conditions provides essential insights into how individuals cope with climatic challenges and maintain homeostasis. Among domestic ruminants, cattle are particularly vulnerable to fluctuations in T and humidity because their thermoregulatory capacity are influenced by behavioral and physiological mechanisms that can be impaired under heat stress (dos Santos et al. 2021). The Temperature–Humidity Index (THI) is used widely as an integrative indicator of environmental heat load, with thresholds commonly cited to classify conditions from comfortable (≤ 70) to stressful (75–78), and > 78 associated with severe distress and a reduced capacity to maintain thermoregulation or normal body T (McDowell et al. 1976). For cattle, some have suggested that mild heat stress begins at a THI of 72, at 79 for moderate stress, and at 89 for severe stress (Yousef 1985). Although those thresholds are well documented in dairy and beef cattle, their physiological implications for Lidia cattle, a rustic and behaviorally distinctive Spanish breed, remain virtually uninvestigated.

The Lidia cattle population in Spain is estimated at around 148,242 animals distributed across approximately 894 breeding farms. The breed is mainly raised in the traditional *dehesa* ecosystems of southwestern and central Spain, particularly in Andalusia, Castilla y León, Extremadura, and Castilla-La Mancha, where the largest concentrations of herds are located (MAPA 2024). Lidia cattle are reared under extensive or semi-extensive conditions that favor natural social structures (e.g., hierarchies and territoriality) and involve limited human contact (Fernández 2005). Such management, coupled with selection on ethological traits, supports distinctive behavioral and autonomic profiles that differ from those of conventional breeds (Lomillos and Alonso 2019). Although Lidia cattle are resilient to environmental stressors, they frequently face abrupt summer heat events that can exceed upper critical limits (García et al. 2018). As such, an understanding of how Lidia cattle physiologically respond to natural thermal variations is essential for evaluations of animal welfare and establishing baseline references that can inform management practices and breed conservation under differing climatic scenarios.

Lidia cattle are managed under extensive conditions with minimal human contact. Their strong temperament and high reactivity make them physiologically and behaviorally comparable to wild ungulates. These characteristics complicate direct physiological assessment and management studies (Gaudioso et al. 1987). Conventional monitoring methods that require restraint or close human presence are difficult to apply and may alter variables such as body temperature (T), heart rate (HR), and heart rate variability (HRV). As a result, reliable field data on autonomic and thermal regulation under natural conditions remain limited. Biologging technology has represented a major methodological advance for studying free-ranging animals. The development of miniaturized implantable sensors now allows continuous recording of internal body temperature and ECG-derived cardiac activity (Ropert-Coudert and Wilson 2005). Subcutaneous biologgers are especially useful in veterinary and wildlife research, where external devices are impractical or may alter natural behaviour. By reducing handling stress, they allow long-term monitoring of physiological responses in free-ranging animals and provide valuable information on environmental and autonomic dynamics (Abecia et al. 2025).

Although biologgers are widely used in wildlife ecology, their application in traditional cattle breeds remains limited, particularly in Lidia cattle, which represent a valuable model for studying physiological adaptations in semi-wild bovines. Continuous monitoring of variables such as heart rate (HR) and especially heart rate variability (HRV) provides a reliable and minimally intrusive method to assess stress responses in real time (von Borell et al. 2007; Kovács et al. 2014). HRV, defined as the variation between consecutive heartbeats, reflects the balance between the sympathetic and parasympathetic branches of the autonomic nervous system. Higher HRV values indicate greater autonomic flexibility and a better capacity to cope with environmental or physiological stressors (Porges 2007).

Despite extensive use of biologgers in production and wildlife species, published accounts of their use with Lidia cattle are unavailable. Our understanding of the behavior of those animals, which have been subjected to genetic selection processes that are very different from those used with other cattle breeds (Cañón et al. 2008), essentially has been based on ethological patterns (Lomillos et al. 2013), and the animals exhibit a unique temperament and stress-coping traits that might modulate their autonomic and thermal responses to environmental heat load. Furthermore, the dynamic interactions between meteorological conditions and physiological outputs over short time-scales have not been quantified in this breed.

The aim of this study was to evaluate the relationships between environmental conditions and physiological responses in Lidia cattle using biologger technology. Specifically, we continuously monitored body T, HR, and HRV in freely moving animals under extensive management conditions. These measurements allowed us to examine how changes in environmental variables are associated with short-term physiological adjustments in this breed.

## Materials and methods

**Fig. 1:**
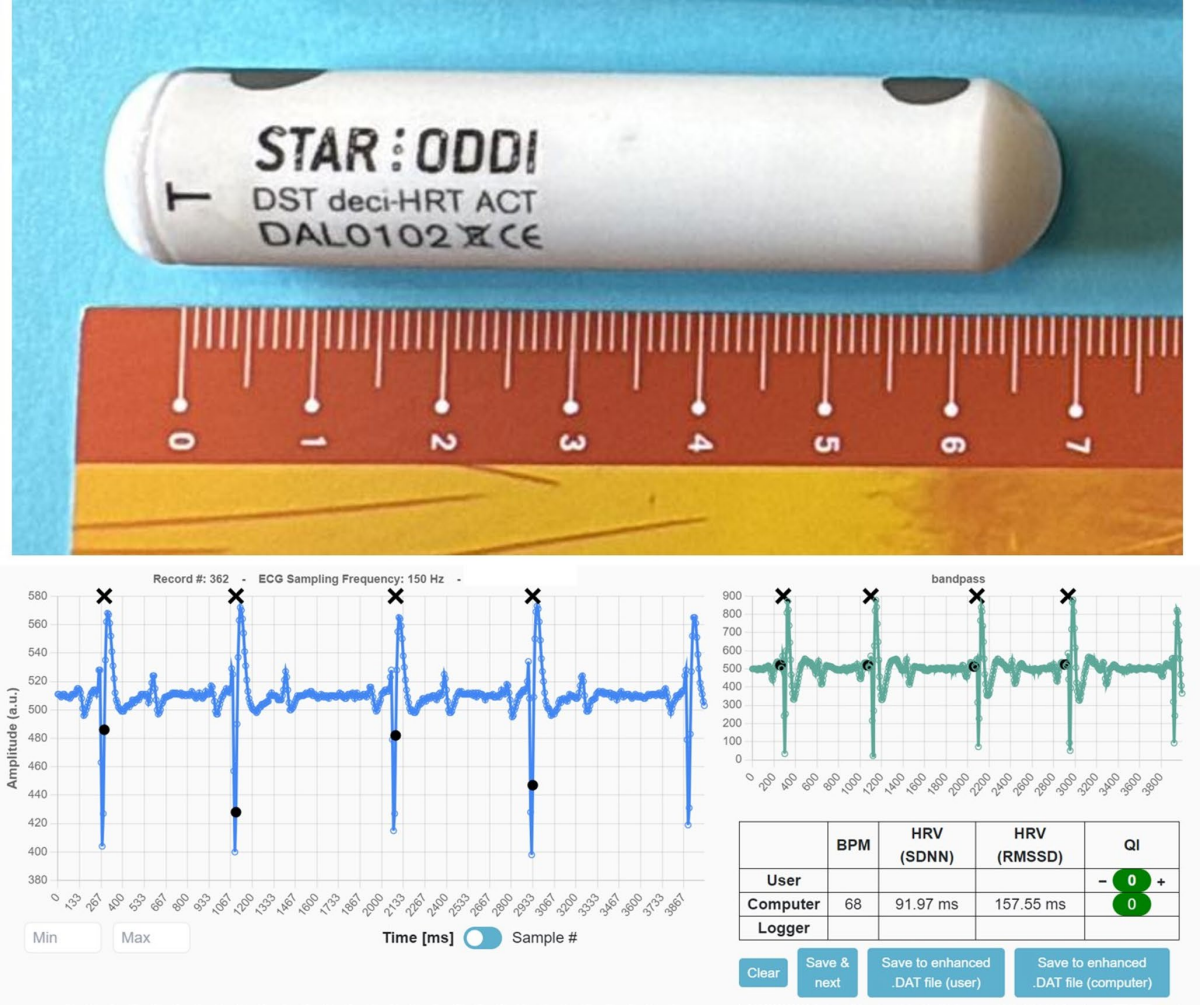
Biologgers used in the study to record body temperature, heart rate and heart rate variability in Lidia cattle (DST deci-HRT ACT, Star-Oddi, Iceland; 15 × 70 mm, 27 g), and an example of a ECG obtained from the HR software

All procedures were approved by the Animal Experimentation Ethics Committee (OEBA; Authorized Body-OH) of the University of León (OEBA-ULE-009-2025). The study was conducted during the second fortnight in June in a *Bos taurus* Lidia cattle herd (Murube lineage) in the province of Salamanca, Spain (40°N). Four adult cows received a subcutaneous biologger (DST deci-HRT ACT, Star-Oddi, Iceland; 15 × 70 mm, 27 g) (Fig. 1) that was designed to record body T and electrocardiogram (ECG)-derived HR and HRV continuously under natural conditions for 10 days (18–27 Jun). The animals used in the study had a mean age of 3 yrs (± 6 mo), and none of the females were pregnant during the experimental period.

The farm comprised three contiguous owned estates and one adjoining leased property, each covering approximately 300 ha. The Lidia cattle herd included 91 cows and 3 breeding bulls. The pastureland was a cold *dehesa* ecosystem that was predominantly holm oak (*Quercus ilex*), but had a small area of oak woodland (*Quercus robur*) near the river. Primarily, the feeding regimen was based on natural pasture and, in periods of low forage availability supplemented by compound feed blocks and straw provided *ad libitum*. Each estate included several natural ponds and a borehole, which ensured a continuous water supply year-round. In addition to the Lidia herd, the farm maintained a population of extensively managed suckler cows for beef production, including Limousin, Charolais, Morucha, and Berrenda en Colorado breeds, and crossbred animals. The total herd comprised approximately 700 cows, which were kept in groups of 50–70 females, each accompanied by one or two breeding bulls.

### Implantation procedure

Animals were restrained in a handling chute and lightly sedated by 23.3 mg of xylazine hydrochloride (Xilagesic^®^, Calier S.A.). The left forelimb was elevated to expose the thoracic region, which was cleaned with povidone-iodine soap solution. Local anesthesia (2 mL, subcutaneous) was administered before a small incision was made in the skin over the left hemithorax. A subcutaneous pocket was created to house the biologger, which had been sterilized by immersion in 0.55% ortho-phthalaldehyde solution for 24 h. The device was placed parallel to the heart axis, and the electrodes were in contact with the underlying muscular layer. The pocket was closed by absorbable sutures, and the incision sealed by an intradermal absorbable suture and sprayed with chlorotetracycline and patent blue (Pederol^®^, Laboratorios Syva, S.A.). Devices were retrieved after the animals were slaughtered at the end of the experiment.

### Sensors and data collection

Biologgers were programmed by Mercury software (v5.83, Star-Oddi, Iceland) to record data every 5 min. The monitoring period was 10 consecutive days during which the animals remained undisturbed in their home pasture. No human interference occurred at that time, except for daily remote visual inspections to ensure the animals’ welfare.

T and HR data were extracted and analyzed to calculate mean (± SE) values for each 24-h period. To identify diurnal and nocturnal fluctuations in physiological activity, circadian patterns were evaluated. From the ECG traces, two time-domain indices of HRV were derived by the Star-Oddi HRT Analyzer software (Fig. 1); i.e., the SDNN (standard deviation of normal to normal R-R intervals) and the RMSSD (root mean square of consecutive deviations between normal heartbeats).

$$\mathrm{SDNN}=\sqrt{\frac{1}{N-1}\sum\limits_{i=1}^{N}\left({R\;R_i}-{\overline{R\;R}}\right)^2}\;\;\;\;\;\;\;\;\;\;\;\; {\overline{\text RR}}=\text{mean of RR intervals}$$

$$\mathrm{RMSSD}=\sqrt{\frac{1}{N-1}\sum\limits_{i=1}^{N-1}\left({R\;R_{i+1}}-{{R\;R_i}}\right)^2}$$

The R-R interval is the time between each detected heartbeat, measured from peak to peak (R) on the QRS complex, which is the cardiac contraction (systole) that begins with the Q wave, a negative deviation, and ends with the R wave, a positive (upward) deviation. The S wave is any negative deflection that occurs immediately following the R wave.

The sampling frequency was set at 150 Hz. The algorithm used to calculate the quality index (QI) of the ECG signal operates in two stages. First, it identifies QRS complexes within the recording and computes each R–R interval. If multiple R–R intervals are detected and their variability is less than 20%, the signal is assigned a QI of 0 (excellent). If no R–R interval is found, the QI is set to 3, and the HR is assigned a value of 2 bpm. Likewise, if the calculated HR falls outside a predefined range, the QI is classified as 3, which indicates unreliability. Typically, HR values that have QI = 3 are excluded from the analysis. If a single R–R interval is detected, and it represents approximately 45% of the total sampling duration, the QI remains 0 (excellent). Any other situation invokes the second stage of the algorithm.

In this second stage, each potential R wave receives a score based on several characteristics; e.g., amplitude and sharpness. The lower-level threshold (LLT) and the higher-level threshold (HLT) are established, which correspond to the lowest and highest grades, respectively. If LLT and HLT overlap (LLT ≥ HLT), it indicates that all potential R waves have similar quality; therefore, all are included in the BPM calculation, and the QI is assigned a value of 1 (good). Typically, that situation occurs in recordings of good quality that display arrhythmias or more than 20% variation between R–R intervals. Otherwise, only R waves graded above the HLT are used for HR estimation. The QI remains 1 (good) unless some R waves fall between LLT and HLT, in which case the classification becomes uncertain, and QI is set to 2 (fair). If only one R wave exceeds the HLT, the QI is assigned a value of 3 (poor), and HR is calculated as 1 bpm. All recordings with QI values of 2 or 3 were unreliable and, therefore, excluded from further analyses. For each ECG recording, the HRT Analyzer software computed HRV based on the SDNN and RMSSD metrics. After processing, the data were exported as a “.HRV” file for additional analyses.

### Cosinor transformation

Circadian rhythms in T, HR, and HRV were graphed by fitting the time-series measurements of each cow to the cosine curve of a 24-h activity rhythm. Midline Estimating Statistic of Rhythm (MESOR, the average value around which the variable oscillates), amplitude (the difference between the peak and the mean value of a wave), and acrophase (the time of peak activity) were calculated for each variable in each animal.

The circadian variables were computed from the classic cosinor model as follows:

$$\mathrm y\left(\mathrm t\right)=\mathrm M+\mathrm A\cdot\cos\left(\left(2\mathrm\pi/24\right)\left(\mathrm t-\mathrm\varphi\right)\right)$$

where M = mesor, A = amplitude, φ = acrophase, and t = time of day (0–24 h).

After fitting the model by least squares, the Root Mean Square Error (RMSE) of the fit is calculated as follows:

$$\mathrm{RMSE}=\mathrm{sqrt}\;\left(\sum\left(\mathrm{y_i}-\widehat{\mathrm y}\mathrm{_i}\right)^2/\left(\mathrm N-3\right)\right)$$

where N is the number of time points, and 3 accounts for the three fitted variables (mesor, cosine, sine).

Then, the Circadianity Index (CI) and the robustness (R²) of the circadian rhythm were calculated. CI was defined as CI = A / RMSE. High CI values (> 3) indicate a strong and stable rhythmicity (large amplitude, small residual error), and low CI values (0–1) indicate weak or noisy rhythms (small amplitude or poor fit). CI ≈ 1–3 indicate a moderate rhythm.

The R² was calculated from the cosinor model fit as follows:

$$\mathrm R^2=1-\left[\sum\left(\mathrm y\_\mathrm i-\widehat{\mathrm y}\_\mathrm i\right)^2/\sum\left(\mathrm y\_\mathrm i-\overline{\mathrm y}\right)^2\right]$$

where y_i = observed values, ŷ_i = values predicted by the cosinor model, and ȳ = mean of the observed data. R² represents the proportion of total variance in the data that is explained by the fitted 24-hour cosine model. It provides an indication of how well the circadian model captures the observed oscillation. R² < 0.2 means a very weak rhythmicity (no clear 24-h pattern), 0.2–0.5 is a moderate rhythmicity (some structure, moderate fit), 0.5–0.8 indicate a strong rhythmicity (model fits the data well), and R² > 0.8 is a very robust rhythm (clear and stable circadian pattern).

### Meteorological data

Mean ambient T (°C), and mean relative humidity (RH) (%) on each day were obtained from the meteorological station closest to the farm (Ciudad Rodrigo, Salamanca, Spain). The temperature–humidity index (THI) was calculated based on the formula of Mader et al. (2006), as follows:

$$\mathrm{THI}\;(\mathrm{cattle})=\left(0.8\mathrm T\right)+\left[\left(\mathrm{RH}/100\right)\left(\mathrm T-14.4\right)\right]+46.4$$

where T = air temperature (°C) and RH = relative humidity.

THI can be used to estimate the degree of climatic stress, and four cattle heat-stress categories based on the THI values can be identified: < 74 normal heat-stress levels; 74 ≤ THI < 79 alert heat-stress levels; 79 ≤ THI < 84 danger heat-stress levels, and THI ≥ 84 emergency heat-stress levels (Papanastasiou et al. 2025).

### Statistical analyses

Raw biologger data were screened for erroneous or physiologically implausible values before statistical analyses. Occasionally, subcutaneous biologgers can record signal artifacts caused by transient sensor noise or motion interference; therefore, a variable-specific filtering approach was applied; i.e., for body T and HR, a rolling median and a median absolute deviation (MAD) filters were used. Each data point was compared to a 3-point moving median, and values differing by more than 3 × MAD were removed, which effectively eliminated isolated spikes while preserving genuine physiological peaks and troughs.

For HRV indices (SDNN, RMSSD), data were filtered based on the interquartile range (IQR) criterion, which excluded values outside the range 𝑄1–1.5 × 𝐼𝑄𝑅, 𝑄3 + 1.5×𝐼𝑄𝑅. That approach was chosen because HRV data often show asymmetric distributions and high natural variability.

After filtering, each variable was visually inspected to confirm that the temporal trends remained biologically coherent and that no real physiological responses were excluded. All subsequent analyses (means, maxima, minima, and lag estimates) were performed on the filtered dataset. Less than 3% of the records were excluded because of noise or biologger artifacts. The filtered data exhibited smooth, biologically coherent profiles and no abrupt discontinuities, which confirmed the efficiency of the filtering procedure. Extreme values of HRV that exceeded several thousand milliseconds were excluded, and the genuine physiological peaks and troughs were preserved.

Descriptive statistics were calculated for all variables (T, HR, SDNN, and RMSSD). Diurnal and nocturnal values were compared by ANOVA. After calculating the cosinor values, the data were pooled and the mean 24-h cosinor curve for each of the four variables was calculated.

The four THI categories (normal heat-stress, alert heat-stress levels, danger heat-stress, and emergency heat-stress) were codified (0, 1, 2 3, and 4, resp.). No emergency heat-stress conditions were observed in the experiment. Changes in THI category (normal to alert or alert to danger) were identified throughout the continuous hourly time series. For each transition, the data from a ± 6-hour window around the change point was extracted to identify the short-term physiological response. Differences in each physiological variable relative to the event were calculated, and the time lag to the maximum response was calculated for each event. Mean ± standard deviation of those lags were computed for each variable.

## Results

Mean (± SE) T, HR, SDNN, RMSSD and max and min values of each variable, and the day on which they occurred are presented in Table 1. In the Lidia cattle in our study, the daytime T (37.55 ± 0.01 °C) was slightly but significantly (*P* < 0.001) higher than was nighttime T (37.51 ± 0.01 °C), but HR did not differ significantly (63.90 ± 0.11 vs. 64.04 ± 0.12 bpm, resp.). HRV variables were significantly (*P* < 0.001) higher at night than they were in the day (17.29 ± 0.11 vs. 15.72 ± 0.09, and 18.96 ± 0.13 vs. 15.06 ± 0.09 ms, for SDNN and RMSSD, resp.).

**Table 1:** Mean (± SE), maximum, and minimum values of body temperature (T), heart rate (HR), and heart rate variability indices (SDNN and RMSSD) in four Lidia cows based on data collected by subcutaneous biologgers throughout a 10-day monitoring period under natural summer conditions

Variable	Mean	Max (day / time)	Min (day / time)
Body T (°C)	37.54 ± 0.01	38.93 °C 19 − 6 / 17:40	33.87 °C 27 − 6 / 07:44
HR (bpm)	63.95 ± 0.08	100 bpm 19 − 6 / 01:30	41 bpm 26 − 6 / 10:52
SDNN (ms)	16.31 ± 0.07	44.42 ms 25 − 6 / 02:10	1.92 ms 18 − 6 / 14:35
RMSSD (ms)	16.53 ± 0.08	48.09 ms 25 − 6 / 09:24	2.10 ms 18 − 6 / 21:10

**Fig. 2:**
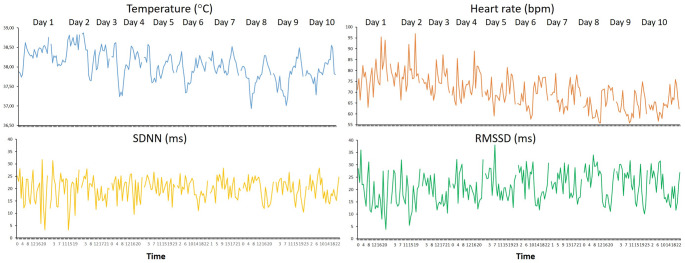
Ten-day continuous time series data of physiological variables recorded in a representative Lidia cow by a subcutaneous biologger under natural conditions in summer. Panels show body temperature (°C), heart rate (bpm), and two time-domain indices of heart rate variability (SDNN and RMSSD, ms)

**Fig. 3:**
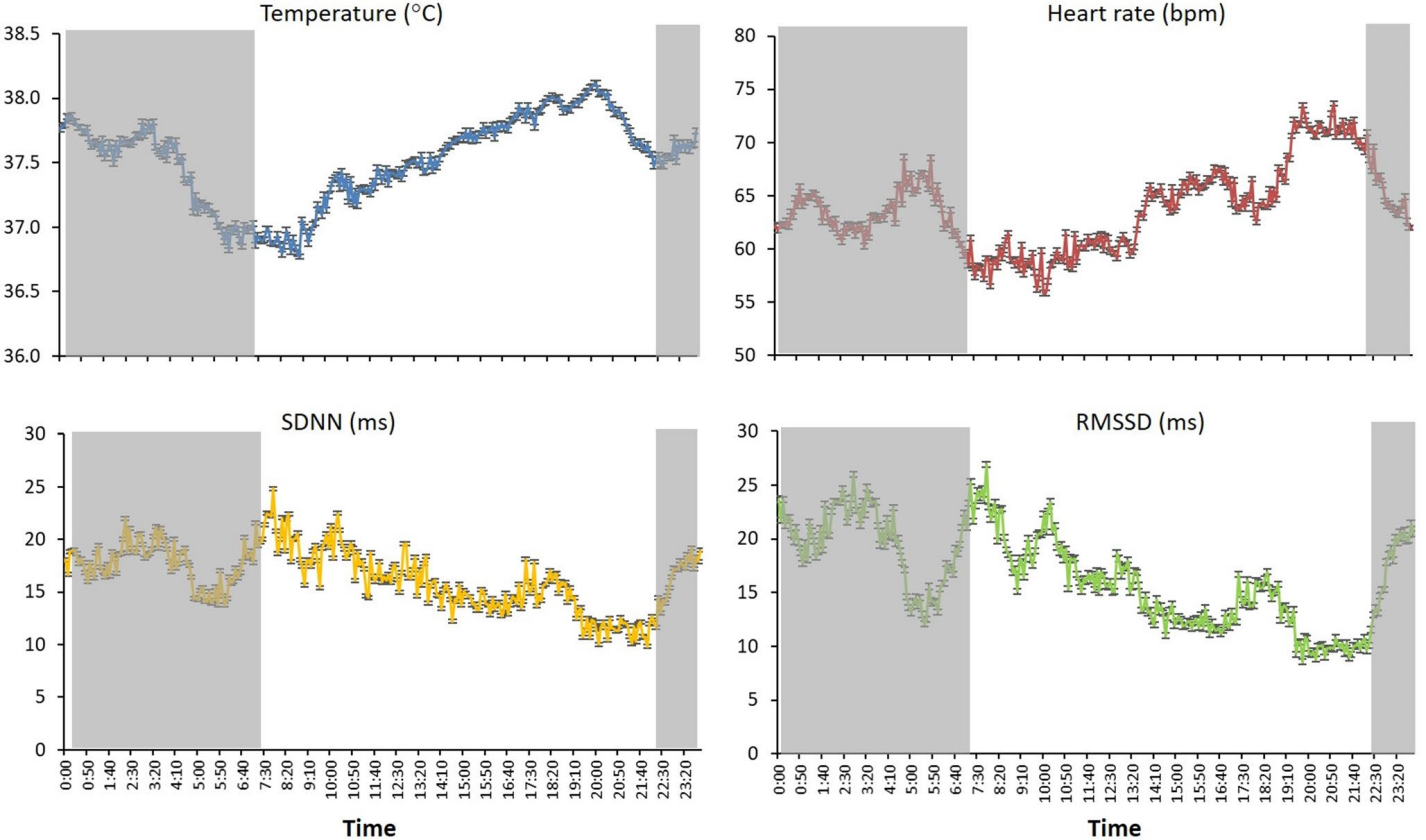
Mean (± SE) body temperature (°C), heart rate (bpm), and heart rate variability indices (SDNN and RMSSD, ms) at 5-min intervals, recorded in four Lidia cows by subcutaneous biologgers under natural conditions in summer. Shaded areas indicate the nocturnal phase

Figure 2 presents the temporal changes in T, HR, SDNN, and RMSSD in a representative cow throughout the 10 days of the study. Mean (± SE) values of the four variables at 5-min intervals are shown in Fig. 3. Body T displayed a clear pattern, with lower values at night and a gradual increase throughout the day, peaking in the evening at around 38 °C. HR exhibited a similar trend, remaining relatively low at night (approx. 60 bpm) and increasing progressively throughout the day, reaching peak values of approximately 75 bpm in the late afternoon or evening. That rise coincided with the increase in body T. Both SDNN and RMSSD were higher at night and decreased throughout the day. SDNN remained relatively stable overnight, but declined gradually throughout the day, which suggested a reduction in autonomic variability under daytime conditions. RMSSD showed a similar pattern, with the highest values at night and a marked reduction throughout the day, which indicated greater parasympathetic dominance at night and an increase in sympathetic activation during waking hours.

**Fig. 4:**
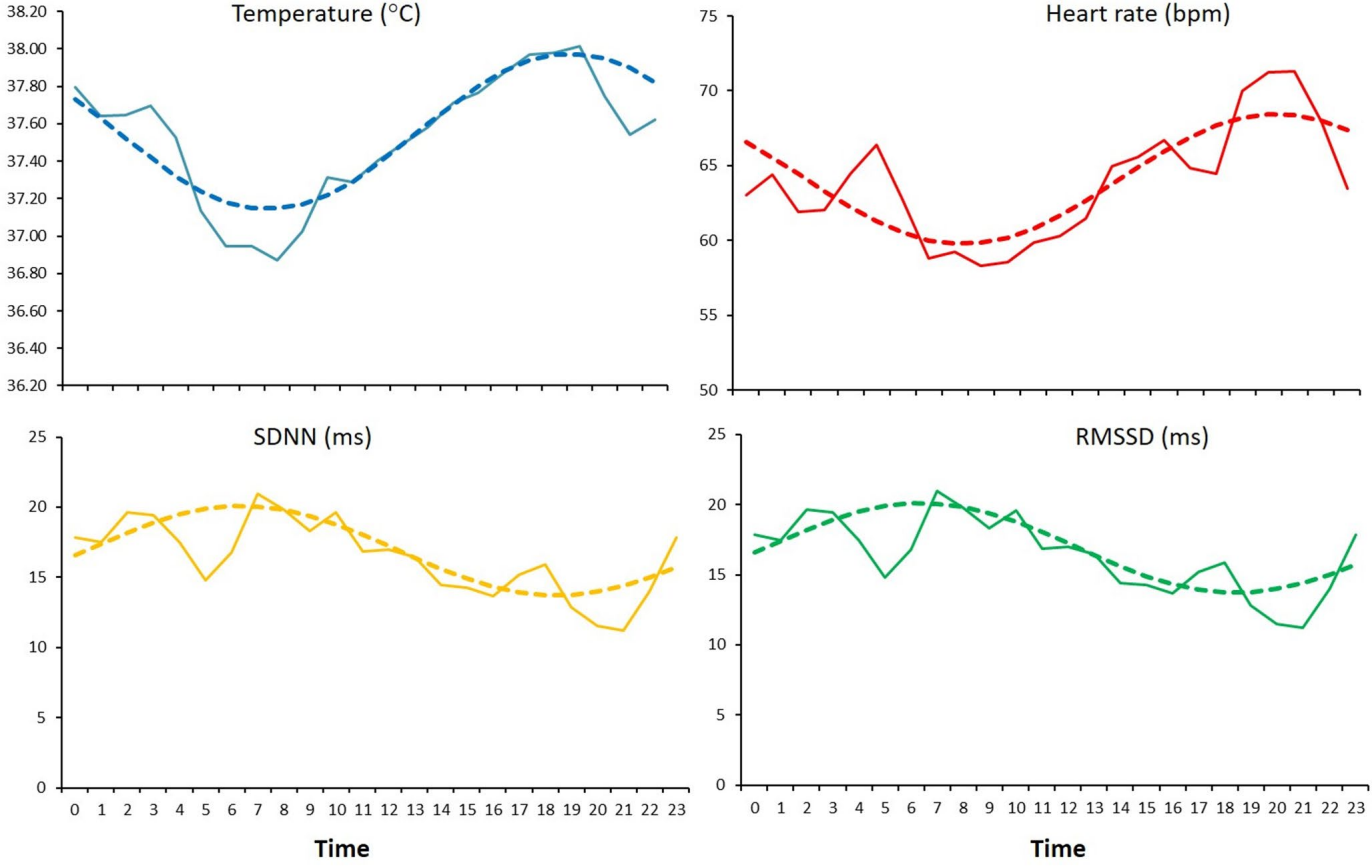
Mean hourly values (solid lines) and fitted 24-hour cosinor curves (dashed lines) for body temperature (°C), heart rate (bpm), and heart rate variability indices (SDNN and RMSSD, ms) in four Lidia cows recorded by subcutaneous biologgers under natural conditions in summer

The circadian analysis of the physiological variables revealed clear rhythmic patterns over the 24-hour cycle (Fig. 4). The results derived from the cosinor fitting are summarized in Table 2. Body T showed a mean acrophase at 1939 h, which indicated a peak in the evening. The rhythm exhibited high robustness and a CI > 3, which reflected a well-defined, stable circadian pattern. The acrophase of HR occurred at 2019 h, close to that of body T, which indicated temporal synchrony between these variables; however, its R^2^ was moderate and the CI ≈ 2, which suggested greater inter-individual variability or external modulation.

**Table 2:** Mean (± SE) circadian rhythm variables (MESOR, amplitude, acrophase, robustness (R²), and circadianity index (CI) of body temperature (T), heart rate (HR), and heart rate variability indices (SDNN and RMSSD) in four Lidia cows based on data recorded by subcutaneous biologgers throughout a 10-day period in summer

	Body T (°C)	HR (bpm)	SDNN (ms)	RMSSD (ms)
MESOR	37.56 ± 0.03	64.08 ± 0.50	16.91 ± 0.43	17.36 ± 0.66
Amplitude	0.42 ± 0.04	4.36 ± 0.73	3.22 ± 0.69	4.91 ± 1.07
Acrophase	19.66 ± 0.35	20.32 ± 0.60	6.38 ± 0.63	5.32 ± 0.80
R^2^	0.84 ± 0.06	0.65 ± 0.13	0.59 ± 0.14	0.58 ± 0.14
CI	3.40 ± 0.84	2.02 ± 0.59	1.73 ± 0.60	1.72 ± 0.61

The HRV indices (SDNN and RMSSD) displayed acrophases in the morning (0623 h and 0519 h, resp.), which indicated peak variability in the early hours of the day. Both metrics had moderate R^2^ and low CI, which suggested weaker or less consistent rhythms than in T and HR.

**Fig. 5:**
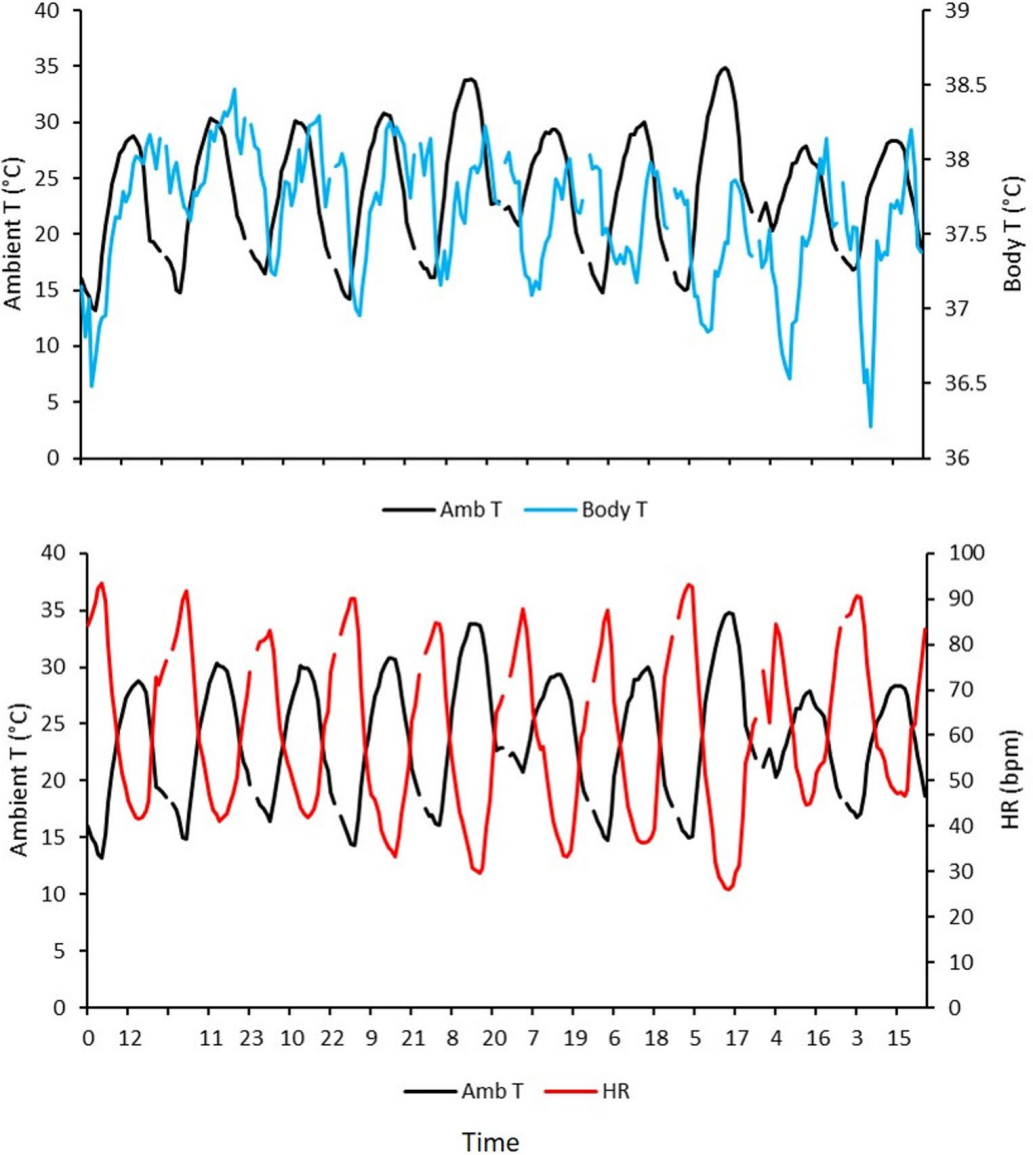
Parallel changes in ambient temperature (black line) with body temperature (upper panel, blue line) and heart rate (lower panel, red line) in four Lidia cows recorded continuously by subcutaneous biologgers for 10 d under natural conditions in summer

In the 10-d period, mean ambient T and RH were 23.9 ± 5.4 °C and 58.2 ± 14.7%, respectively. The changes in ambient T paralleled those in body T and HR (Fig. 5). Both variables showed similar daily oscillations, and ambient T exhibited the widest fluctuations (ranging from approximately 10 °C to 38 °C). The pattern indicated that body T followed ambient variations with limited amplitude, which suggested an effective thermoregulatory capacity that buffers rapid environmental changes. The amplitude of HR changes (from ~ 40 bpm at night to ~ 90 bpm during heat peaks) demonstrated the cardiovascular responsiveness of the animals to thermal load. HR tended to rise slightly after or in synchrony with peaks in ambient T.

**Fig. 6:**
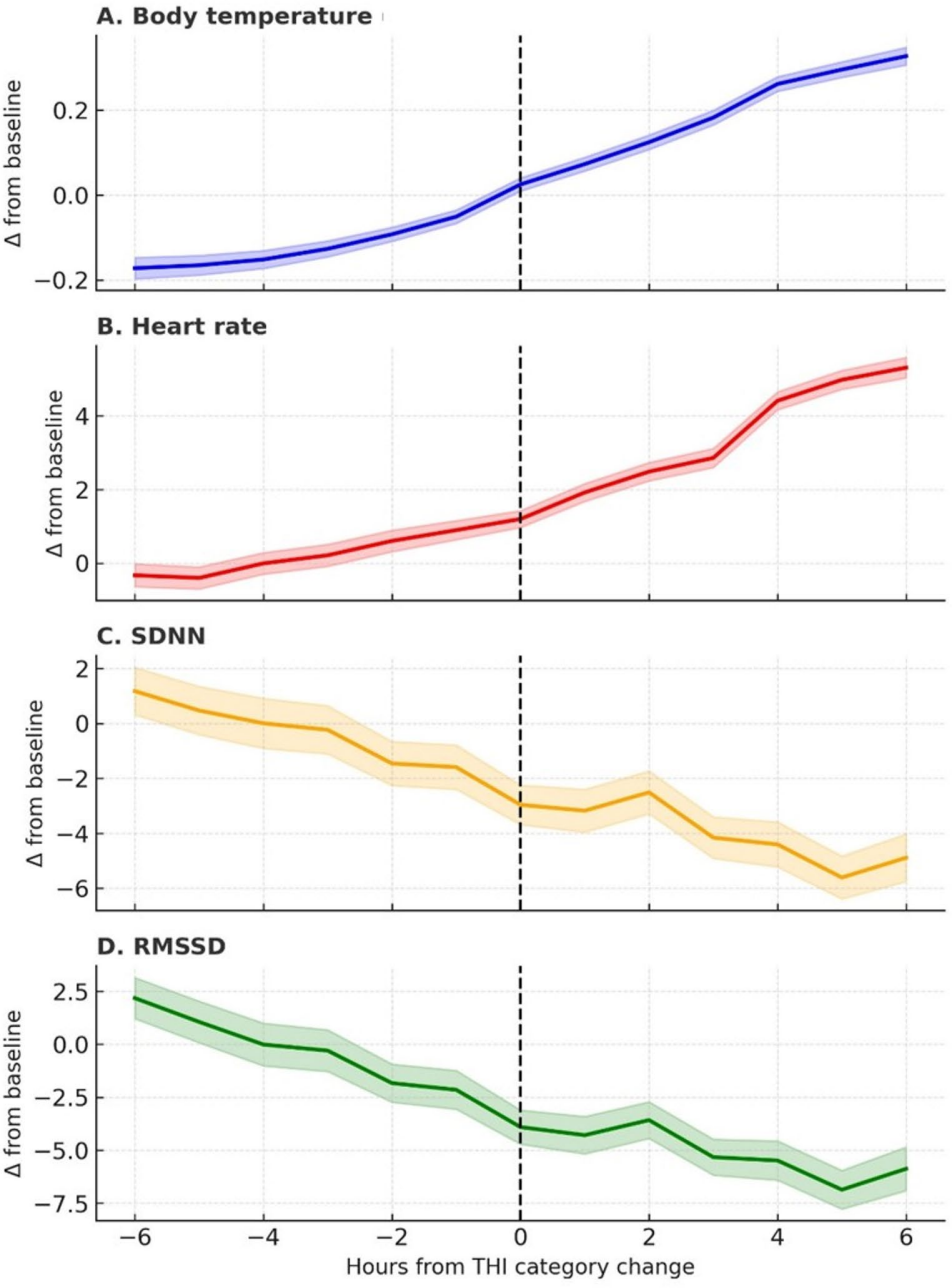
Mean (± SE) changes (Δ) in physiological variables in Lidia cows relative to baseline (0 h) following a change in the Temperature–Humidity Index (THI) category. Panels show (**A**) body temperature, (**B**) heart rate, (**C**) SDNN, and (**D**) RMSSD, recorded by subcutaneous biologgers under natural conditions in summer. Vertical dashed lines indicate the moment of the change in the THI category

A slight delay occurred between peaks in ambient T and increases in body T and HR, which indicated that thermoregulatory adjustments in Lidia cows are not instantaneous; rather, they occur gradually as heat accumulates in body tissues. That pattern was consistent with the quantitative lag analysis (Fig. 6). There were temporal changes in the physiological responses following a change in the THI (± 6 h window), relative to the baseline value at the moment of a change in the level of the THI. Body T and HR reached their maximum increases 6 h after an increase in THI, and HRV indices (SDNN and RMSSD) were lowest at about 5 h post-event.

The detailed temporal analysis of the physiological absolute values (not the relative curves) revealed that each variable responded with a distinct time lag following transitions in heat stress intensity (THI category changes). Body T showed a gradual increase, which, on average, was at its maximum 4.49 ± 0.24 h after the change in the THI. HR responded more rapidly and, on average, peaked 3.05 ± 0.24 h after the increase in the THI. SDNN was lowest 3.91 ± 0.22 h after the THI transition, and RMSSD was, on average, decreased to its lowest 3.66 ± 0.22 h after the change in THI, which is consistent with parasympathetic withdrawal and an increase in physiological strain.

## Discussion

This study is the first to provide results on the continuous physiological monitoring of Lidia cattle under natural environmental conditions based on data from subcutaneous biologgers. The results have demonstrated that this cattle breed exhibits distinct circadian patterns in T, HR, and HRV, which are strongly associated with ambient T and thermal humidity index (THI). As in our study, other studies that used the same biologgers in beef cattle breeds in Spain (Palacios et al. 2021) and in Hereford, Jersey, Norwegian Red breeds, and their crosses in Norway (Niccolai et al. 2024), documented circadian patterns in body T, HR, and locomotor activity. The combination of continuous physiological and meteorological data has provided novel insights into the thermoregulatory resilience and autonomic flexibility of Lidia cattle.

In Lidia cattle, body T and HR exhibited well-defined diurnal fluctuations; viz., they increased in the day and decreased at night. Similarly, other studies have shown that, in domestic cattle, typically, body T peaks in the late afternoon because of increased metabolic activity and heat accumulation (Piccione et al. 2003; Palacios et al. 2021; Suarez-Trujillo et al. 2022). Lomillos et al. (2017) demonstrated that Lidia cows maintained under extensive *dehesa* conditions exhibited well- defined circadian activity rhythms and resting periods based on data collected by GPS–GPRS technology. That study revealed that those animals began their grazing activity before dawn, reduced activity in the warmest hours of the day, and displayed a seven-hour nocturnal rest phase, which indicated a strong synchronization between behavior and environmental cycles. Such findings reinforce the idea that Lidia cattle possess adaptive traits that allow them to maintain stable behavioral and physiological rhythms despite large diurnal thermal fluctuations (Lomillos et al. 2017).

In our study, HRV indices (SDNN and RMSSD) were higher at night than they were during the day, which indicated an increase in parasympathetic tone during rest and the dominance of sympathetic activity during periods of high environmental heat load. Such reciprocal dynamics between HR and HRV are widely recognized as markers of autonomic balance in cattle (von Borell et al. 2007; Kovács et al. 2014). The decline in HRV under heat stress reflects reduced vagal modulation and enhanced sympathetic activation, which is an adaptive mechanism that allows the maintenance of cardiovascular output and body temperature within physiological limits (Kitajima et al. 2021).

The R² and CI derived from the cosinor model provide complementary insights into the stability and strength of biological rhythms in cattle. In our study, body T exhibited a high R² (> 0.8) and CI (> 3), which reflected a consistent, well-defined circadian oscillation. Such stability under fluctuating outdoor conditions suggests strong endogenous control and efficient entrainment to environmental cues such as light–dark cycles and changes in ambient temperature (Piccione et al. 2003; Refinetti 2016). In contrast, HR and HRV indices had lower R² and CI, which indicated a weaker or more variable rhythmicity and probably reflected a strong influence of transient autonomic adjustments and behavioral modulation (Kovács et al. 2014). The CI, which combines the amplitude and residual error of the cosine fit, has been proposed as a sensitive indicator of rhythm coherence and physiological resilience under stress (Refinetti 2016). The moderate CI for the HRV indices in Lidia cows might reflect the combined effects of environmental variation and the breed’s strong behavioral reactivity. Studies of dairy cows under thermal and management stress have linked reduced rhythm robustness and CI to circadian disruption and autonomic imbalance (Suarez-Trujillo et al. 2022; Montes et al. 2023). The coexistence of a robust thermal rhythm and a moderately stable cardiac rhythmicity in this breed supports the hypothesis that Lidia cows maintain thermoregulatory homeostasis even if autonomic flexibility is transiently reduced under heat load.

In our study, there was a temporal delay between changes in ambient T and physiological responses. Body T rose approximately 4.5 h after an increase in the THI, and HR peaked about 3 h post-change in THI. That lag suggests that cardiac activation precedes thermal adjustment, which is an anticipatory response to an increase in heat load (Lees et al. 2020). Such hierarchical responses are consistent with classical thermophysiological models in cattle, where HR modulation and peripheral vasodilation precede measurable rises in core T (Mader et al. 2006; Polsky and von Keyserlingk 2017).

The decline in HRV following increases in the THI reflects a delay in autonomic recovery after sympathetic activation. In dairy and beef cattle that have been exposed to acute heat stress, autonomic restoration lags behind T normalization (Frese et al. 2017; Kitajima et al. 2021). Together, those temporal dynamics underscore the multi-faceted nature of thermal adaptation, integrating cardiovascular, neural, and thermoeffector mechanisms operating on different timescales.

Biologgers have provided a methodological breakthrough for the study of behaviorally reactive cattle such as the Lidia breed. Traditional monitoring techniques that involve manual restraint or sporadic sampling are impractical and can alter physiological responses because the cause handling stress (Mohr et al. 2002; von Borell et al. 2007). By contrast, subcutaneous biologgers provide continuous, high-resolution measurements of body T and ECG-derived HR and HRV, which allows the detection of subtle physiological shifts without human interference (Ropert-Coudert and Wilson 2005; Brown et al. 2013). Recent studies that have used the same biologgers in other cattle breeds have demonstrated their reliability for long-term monitoring and welfare assessment (Palacios et al. 2021; Niccolai et al. 2024). The use of those devices to monitor Lidia cows, which are kept under extensive conditions and experience minimal human contact, has shown that precise physiological monitoring can be extended beyond conventional livestock to include rustic and traditional breeds. That approach provides new opportunities for assessing adaptation, welfare, and resilience under natural and management-induced stressors.

In our study, despite exposure to pronounced thermal variations, Lidia cattle maintained relatively stable body T and HRV patterns, which reflected high thermoregulatory efficiency. That buffering capacity suggests that those animals possess effective physiological and behavioral adaptations to heat, which likely are derived from centuries of selection in Mediterranean environments. Similar resilience has been reported in zebu and Creole breeds that have been exposed to natural heat stress, which suggests that rustic genotypes have a higher thermotolerance than do highly selected dairy breeds (Lees et al. 2020).

From an welfare perspective, an understanding of the physiological thresholds of heat stress in extensive systems is essential. The combination of biologging and environmental data provides an early warning tool for identifying periods of thermal strain, which can enable targeted management interventions such as providing shade or modifying grazing schedules (Polsky and von Keyserlingk 2017; Brown et al. 2013). Furthermore, the capacity to monitor HRV continuously offers a non-invasive indicator of autonomic balance and welfare state, which is superior to conventional behavioral assessments.

## Conclusions

Our study has demonstrated that biologging technology offers unprecedented opportunities to investigate the physiological ecology of a behaviorally reactive cattle breed. The continuous recording of thermal and cardiac variables under free-ranging conditions revealed that Lidia cattle exhibit well-defined circadian rhythms and delayed but coordinated physiological responses to environmental heat stress. Those results highlight the importance of integrating real-time physiological data into welfare and conservation frameworks, especially in the context of global climate change. Future research should expand this approach to larger populations and longer periods to establish reference thresholds for heat tolerance and autonomic resilience among multiple cattle breeds and environmental settings. In addition, this study has demonstrated the value of Lidia cattle as a model for studying physiological adaptations in extensive systems. Their rusticity and minimal human interactions make them a key breed for understanding how cattle maintain homeostasis in the face of thermal variation, which can provide a useful basis for future comparisons with other breeds and for improving management strategies under heat stress.

## Data Availability

Data will be made available on request.
